# Validation and optimization of the French Generic Adherence for Chronic Diseases Profile (GACID-P) using classical test and item response theory

**DOI:** 10.1186/s12955-023-02130-0

**Published:** 2023-05-24

**Authors:** Christine Rotonda, F. Guillemin, T Conroy, C. Alleyrat, B. Lefevre, M. Soudant, C. Tarquinio

**Affiliations:** 1 Université de Lorraine, Centre Pierre Janet, Metz, F-57000 France; 2 Université de Lorraine, APEMAC, équipe EPSAM, Metz, F-57000 France; 3 CHRU Nancy, Inserm, Université de Lorraine, CIC, Epidémiologie Clinique, Nancy, F-54000 France; 4 Université de Lorraine, APEMAC, équipe MICS, Nancy, F-54000 France; 5 Institut de Cancérologie de Lorraine, Department of Medical Oncology, Vandoeuvre- lès-Nancy, F-54519 France; 6 CHRU-Nancy, Université de Lorraine, Service des Maladies Infectieuses et Tropicales, Nancy, F-54000 France

**Keywords:** Adherence profile, Generic, The generic adherence for chronic Diseases Profile, Validation, Item response theory

## Abstract

**Background:**

The Generic Adherence for Chronic Diseases Profile is a French generic scale (GACID-P) developed to measure adherence in several disease areas such as cardiology, rheumatology, diabetes, cancer and infectiology.

**Method:**

We aimed to study the measurement invariance of the Generic Adherence for Chronic Diseases Profile by an item response model, optimize the new instrument version from item response model and qualitative content analyses results, and validate the instrument. The metric properties of the optimized version were studied according to classical test theory and item response model analysis.

**Results:**

A sample of 397 patients consulting at two French hospitals (in diabetes, cardiology, rheumatology, cancerology and infectiology) and in four private practices was recruited; 314 (79%) patients also completed the questionnaire 15 days later. Factor analyses revealed four dimensions: “Forgetting to take medication”, “Intention to comply with treatment”, “Limitation of risk-related consumer habits” and “Healthy lifestyle”. The item response model and content analyses optimized these four dimensions, regrouping 32 items in four dimensions of 25 items, including one item conditioned on tobacco use. The psychometric properties and scale calibration were satisfactory. One score per dimension was calculated as the sum of the items for the dimensions “Forgetting to take medication” and “Intention to comply with treatment” and as a weighted score according to the item response model analysis for the two other dimensions because of differential item functioning found for two items.

**Conclusion:**

Four adherence profile scores were obtained. The instrument validity was documented by a theoretical approach and content analysis. The Generic Adherence for Chronic Diseases Profile is now available for research targeting adherence in a broad perspective.

**Supplementary Information:**

The online version contains supplementary material available at 10.1186/s12955-023-02130-0.

## Introduction

Adherence in the area of chronic disease is a set of behaviours that may include not only medication but also diet and lifestyle behaviours that affect patient health. It is sometimes supplemented with other behaviours that are most often included in the definition of adherence. These behaviours may include coming to hospital or doctor’s office appointments, eating a healthy and balanced diet, exercising, and avoiding smoking [1]. Therefore, in a broad sense, adherence refers to the behaviour of a patient who follows the prescribed treatment, and it takes into account medical recommendations. Haynes defines adherence as “the extent to which an individual’s behaviours (in terms of taking medication, following regimens, or making lifestyle changes) coincide with medical or health advice”[2]. Thus, adherence should be seen as a process of patient adherence to physician suggestions. It is not a process of submission but rather an approach of a transactional nature between physicians and patients that promotes reciprocal adjustments.

Poor adherence is associated with increased morbidity and mortality as well as increased health care costs [3–5]. This association has been particularly demonstrated in several areas. In cardiology, for example, Mazzaglia et al. followed 18,806 newly diagnosed hypertensive patients without heart disease for 5 years at the time of inclusion [3]. Non-adherence (defined as the total number of days’ supply of medication dispensed divided by the length of the corresponding follow-up and multiplied by 100) ) was the benchmark. The authors found a 38% reduction in number of cardiovascular events in highly adherent patients. Similarly, in a study of 1,076 patients with type 1 diabetes, only 39% measured their blood glucose levels on a daily basis [6]. In a type 2 diabetes study, only 67% of patients tested their glycemia at least once a day [7]. In rheumatology, adherence is also a problem that needs to be addressed [8]. The problem is especially important in osteoporosis because of its asymptomatic nature and the contrast between poor adherence and the existence of effective therapies. The proportion of adherence to anti-osteoporotic drugs ranges from 43 to 81%, with an average of about 50% within 1 year [9]. A Canadian study in osteoporosis showed that adherence of 50% or less increased the risk fracture up to 40% [9]. In oncology, there is evidence of oral adherence rates as low as 46% for anti-neoplastic drugs [10]. The variability in measurement methods makes comparability within or between conditions difficult or not possible. This variability has often been reported in other areas such as HIV infection [5]. The problem of adherence in the field of cancer [11] is still recent in contrast to other chronic diseases such as diabetes [7, 12] or heart disease [3, 13].

Paradoxically, the importance of taking adherence into account in managing chronic diseases is no longer doubted, but its measurement remains problematic. Two types of measures are generally used: direct and indirect [5, 14, 15]. Direct measures involve using biological markers present in the organism. These are supposed to indicate whether or not the patient has followed medical prescriptions. These measurements are objective but are intrusive, expensive, impossible to implement routinely and not always reliable. Indirect measurement methods, although less “objective” than direct methods, seem more easily applicable by practitioners and clinicians [16]. The main techniques are the self-reporting questionnaire, patient interview, hetero-questionnaire, electronic pill dispenser, drug count, follow-up notebook, and honoured appointments. The most frequently used indirect methods are the questionnaire and semi-directive interview [17]. The Morisky Medication-Taking Adherence Scale (MMAS) classifies patients according to three adherence profiles (good, mediocre or poor) [18]. In cancer, a review of the literature showed that measures of adherence were heterogeneous and empirical (hair tests, interviews, drug counts, more or less elaborate questionnaires, etc.) [19].

However, to date, no generic scale for measuring adherence is available, even though the problem of therapeutic adherence appears in many chronic diseases. In daily practice, such a tool would allow for identifying patients with little or no adherence, to provide more appropriate care in the context of therapeutic education programmes, for example, the aim of which is often and precisely to improve adherence. Poor adherence to treatment can also lead to reduced effectiveness of treatments (particularly long-term treatments) or to overdoses due to excessive intake, which can lead to complications and costly hospitalisation.

We aimed to develop and validate a generic adherence scale adapted to several disease areas such as cardiology, rheumatology, diabetes, cancer and infectiology by using classical test and item response theory (IRT).

## Method

### Development of the generic adherence for chronic diseases profile (GACID-P) questionnaire

A review of the English and French literature on the development or validation of adherence questionnaires published since 1980 identified 20 published and validated scales comprising a total of 330 items. This item bank was used to create new items for our tool (either by adapting some items from this bank or by creating new items as for majority of items). After a content analysis by a group of experts (psychologists, clinicians, nurses), these items were grouped together in three dimensions: (1) medication and/or medical adherence: adherence to medical prescriptions in terms of dose, schedules, attending medical appointments, tests (blood test, X-ray, etc.) requested by the doctor; (2) lifestyle adherence; (3) diet adherence: sometimes optimized care is achieved by following a restrictive diet or avoiding excess (sugars, fats, etc.). The purpose of this step was to bring together dimensions that were scattered in the different questionnaires depending on whether they were created from a clinical, public health or psychological approach. Then, 26 health professionals from university hospitals (8 cardiologists, 5 diabetologists, 3 rheumatologists, 4 nurses, 6 health psychologists) and 9 general practitioners of the Grand-Est region in France divided into 5 multiple health-professional groups, grouped items (among the 330) with the same meaning. The items with the clearest or most accurate wording were retained in each group, or the items were reworded for more relevance in terms of measured adherence, which resulted in a corpus of 41 items. An expert group of seven health professionals with specific knowledge of adherence (one general practitioner, one cardiologist, one diabetologist, one rheumatologist, one nurse, two health psychologists) then reduced this list to 32 items. The instructions were to cover all three dimensions of adherence identified in the literature review, ensure that the items were consistent and congruent for all medical specialties (and adjust their wording accordingly), and avoid unnecessary repetition and retain items that were relevant. A final phase consisted of a cognitive debriefing with focus groups of patients to guarantee good understanding of the 32 items and, if necessary, adapt the reformulation. Four focus groups of six patients with various conditions (heart disease, diabetes, rheumatic diseases, cancer, others) reviewed all 32 items to ensure that they were well understood and appropriate to the reality of the disease as experienced by the patients.

### Study sample

The sample consisted of patients with chronic disease consulting at two university hospitals (during consultations in diabetology, cardiology, rheumatology, oncology and infectiology) and in three private practices (diabetology, cardiology, rheumatology) of the Grand-Est region, France. The inclusion criteria were (1) age > 18 years; (2) in routine consultation; and (3) able to complete the questionnaire. A specialist clinician confirmed the inclusion criteria for each condition: (1) chronic cardiovascular diseases such as heart failure, hypertension and coronary heart disease; (2) type 2 diabetes; (3) knee or hip osteoarthritis, osteoporosis, inflammatory rheumatism (rheumatoid arthritis and spondyloarthropathy); (4) consulting in a hospital clinic and receiving oral non-hormonal anti-cancer drugs; and (5) consulting in a hospital clinic and living with HIV infection. We excluded patients not receiving any treatment (drug or lifestyle/dietary recommendations) as well as those with more than three conditions cited in the inclusion criteria.

### Measured variables

The GACID-P questionnaire is a French adherence questionnaire consisting of 32 items covering three components of health adherence: medication and/or medical adherence (i.e., adherence with medication prescriptions and examinations: items 1 to 22); lifestyle adherence (physical activity, addictive behaviours, etc.: items 23, 24 and 28 to 30) and diet adherence (i.e., limited intake of fat, sugar and salt: items 25 to 27). Some items measure non-adherence to therapy (items 2 and 6 to 16) and others measure adherence to therapy. Responses to the items were rated on a 4-point Likert-type scale (1, never, to 4, all the time).

The 8-item MMAS (MMAS-8) is a generic medication adherence scale [18] validated in French [20]. The total score on the MMAS-8 ranges from 0 to 8, with scores of < 6, 6 to < 8, and 8 reflecting low, medium, and high adherence, respectively.

For each patient included during the consultation, the physician completed a “treatment and history” document specifying the different treatments under way in the previous month as well as the surgical history and comorbidities. Socio-demographic data collected were sex, age, marital status (single, married/ cohabitating, divorced, partnership, widow(er)), education (certificate of studies, certificate of secondary education, technical school certificate, baccalaureate degree (general or professional), post-baccalaureate degree), employment status (full-time, part-time, unemployed, retired).

### Conduct of the study

The physician in one of the 12 participating centres administered the first questionnaires including GACID-P and MMAS-8 scales and socio-demographic and treatment data to the patient during a routine visit after the study was explained to the patient (D0). At the end of this consultation, the patient was given the questionnaires including GACID-P and MMAS-8 scales only in a pre-stamped envelope to be completed 15 days later (D15).

### Statistical analysis

Sociodemographic and clinical characteristics of patients are described with number (%) for categorical variables and number and mean (SD) for quantitative variables. To determine the acceptability of the questionnaire, the distribution of response modalities for the items was analysed, with a search for a floor or ceiling effect as well as the percentage of missing items. Two types of factorial analyses of the questionnaire were performed: principal component analysis (PCA) with rotation for items with polytomous coding and multiple correspondence analysis (MCA) for items with dichotomous coding following study of the distribution of items. The properties of GACID-P questionnaire dimensions identified by PCA and MCA were further studied by using a model of item response by dimension [21], involving a Rasch model for dimensions with dichotomous response items [22] and a partial credit model for dimensions with polytomous items [23]. Item and person fit were tested with standardized residuals (a summation of individual person and item deviations) and as a chi squared statistic. Residual values of items between ± 2.5 are deemed to indicate adequate fit to the model [24] [25]. For participants deviating, underfit means random or constant responses and overfit an attraction for extreme response patterns. Disordered thresholds within an item indicated consistent difficulty in discriminating between response categories. The internal consistency of the dimensions was assessed by the Person Separation Index (PSI), with expected value > 0.85 [26]. According to Tennant et al. “Person Separation Index (PSI) is interpreted in the same way as Cronbach’s alpha. In fact, the only calculation difference between PSI and Cronbach’s alpha lies within the value used within the formula, with PSI using the logit value and Cronbach’s alpha using the raw value. The PSI is an indication of reliability and reflects the ability to differentiate between different levels of the underlying construct” [24, 26]. Local dependency was identified with a residual correlation (r) at least 0.3 higher than the mean of the correlations between pairs of items on the scale [27]. The invariance of the items was studied by differential item functioning (DIF) according to different factors such as the present condition, sex, smoking statusonly available in infectiology patients (smoker, former smoker and non-smoker) and professional status (not working, work part time, work full time). For each item, DIF is detected by comparing graphically and by ANOVA the response probabilities observed between the different levels of the underlying characteristic and between different groups [22]. Finally, following the results of the IRT analyses, a content analysis was carried out in accordance with recommendations for reducing measurement scales [28]. In each dimension defined by factor analyses and optimized by IRT and content analyses, the internal consistency was determined by calculating Cronbach’s alpha, with a value ≥ 0.7 considered acceptable [29]. The reproducibility of the dimensions was studied between D0 and D15 by calculating the intra-class correlation coefficient (ICC). A value 0.6 to 0.8 was considered good and > 0.8 excellent [30]. The convergent validity of the instrument was assessed by Spearman correlation analysis of scores for the dimensions of the GACID-P questionnaire on drug adherence and the MMAS-8 score.

Statistical analysis involved use of SAS v9.4 (SAS Inst., Cary, NC) for classical test theory analysis and RUMM2030 (Rumm Laboratory, Perth, Western Australia) for IRT analysis. All p-values were Bonferroni-corrected for IRT analysis and overall significance was set to 0.05 for the other analyses.

## Results

### Description of the sample

**Fig. 1:**
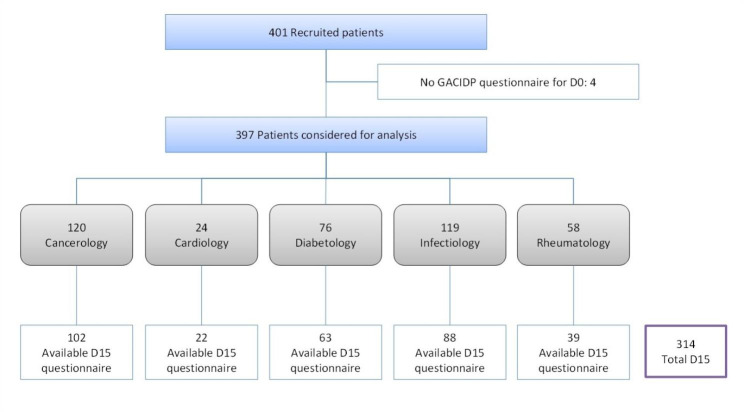
Flowchart of inclusion and follow-up of patients with chronic diseases D0: Days 0; D15: Day 15

A sample of 397 patients was included, and they completed the French GACID-P questionnaire at the inclusion visit; 314 (79.1%) patients completed the questionnaire at D15 (Fig. 1). The mean age was 58 (SD 11.1) years, 53.4% of patients were male, 53.6% were married or living with a partner and 44% were retired. The characteristics of the sample by condition are in Table 1.

**Table 1:** Characteristics of the study population by disease groups

	Disease groups											Total		
	**Oncology**		**Cardiology**		**Diabetes**		**Infectiology**		**Rheumatology**					
	N = 120		N = 24		N = 76		N = 119		N = 58		N = 397			
Sex														
Male	53	(44.5%)	16	(66.7%)	40	(52.6%)	90	(76.3%)	12	(20.7%)	211		(53.4%)	
Female	66	(55.5%)	8	(33.3%)	36	(47.4%)	28	(23.7%)	46	(79.3%)	184		(46.6%)	
Age														
N	114		21		74		111		53		373			
Mean (SD)	60.5 (10.8)		64.5 (9.3)		62.5 (8.2)		51.1 (10.0)		58.9 (12.1)		58.1 (11.2)			
Marital status														
Single	9	(7.6%)	1	(4.3%)	8	(10.5%)	54	(47.0%)	3	(5.3%)	75		(19.2%)	
Married/ cohabitating	76	(63.9%)	19	(82.6%)	50	(65.8%)	32	(27.8%)	32	(56.1%)	209		(53.6%)	
Divorced	10	(8.4%)	0	(0.0%)	6	(7.9%)	15	(13.0%)	8	(14.0%)	39		(10.0%)	
Partnership	10	(8.4%)	1	(4.3%)	4	(5.3%)	10	(8.7%)	8	(14.0%)	33		(8.5%)	
Widow(er)	14	(11.8%)	2	(8.7%)	8	(10.5%)	4	(3.5%)	6	(10.5%)	34		(8.7%)	
Level of education														
Certif. of studies	22	(18.8%)	3	(12.5%)	14	(19.4%)	12	(11.0%)	12	(21.8%)	63		(16.7%)	
Certif. of secondary education	7	(6.0%)	3	(12.5%)	2	(2.8%)	8	(7.3%)	2	(3.6%)	22		(5.8%)	
Technical school certificate	35	(29.9%)	11	(45.8%)	28	(38.9%)	36	(33.0%)	18	(32.7%)	128		(34.0%)	
Baccalaureate degree (general or professional)	18	(15.4%)	1	(4.2%)	7	(9.7%)	16	(14.7%)	8	(14.5%)	50		(13.3%)	
Post-baccalaureate degree	35	(29.9%)	6	(25.0%)	21	(29.2%)	37	(33.9%)	15	(27.3%)	114		(30.2%)	
Employment														
Full-time														
No	102	(85.0%)	21	(87.5%)	66	(86.8%)	67	(56.3%)	43	(74.1%)	299		(75.3%)	
Yes	18	(15.0%)	3	(12.5%)	10	(13.2%)	52	(43.7%)	15	(25.9%)	98		(24.7%)	
Part-time														
No	116	(96.7%)	24	(100.0%)	71	(93.4%)	108	(90.8%)	54	(93.1%)	373		(94.0%)	
Yes	4	(3.3%)	0	(0.0%)	5	(6.6%)	11	(9.2%)	4	(6.9%)	24		(6.0%)	
Unemployed														
No	115	(95.8%)	21	(87.5%)	72	(94.7%)	104	(87.4%)	51	(87.9%)	363		(91.4%)	
Yes	5	(4.2%)	3	(12.5%)	4	(5.3%)	15	(12.6%)	7	(12.1%)	34		(8.6%)	
Retired														
No	56	(46.7%)	7	(29.2%)	24	(31.6%)	101	(84.9%)	33	(56.9%)	221		(55.7%)	
Yes	64	(53.3%)	17	(70.8%)	52	(68.4%)	18	(15.1%)	25	(43.1%)	176		(44.3%)	
Presence of co-morbidities														
Joint diseases	2	(1.7%)	3	(12.5%)	7	(9.2%)	9	(7.6%)	40	(69.0%)	61		(15.4%)	
Osteoporosis	0	(0.0%)	1	(4.2%)	0	(0.0%)	5	(4.2%)	4	(6.9%)	10		(2.5%)	
Arteritis of the lower limbs	6	(5.0%)	2	(8.3%)	6	(7.9%)	3	(2.5%)	0	(0.0%)	17		(4.3%)	
Back pain	0	(0.0%)	0	(0.0%)	11	(14.5%)	8	(6.7%)	11	(19.0%)	30		(7.6%)	
Asthma	1	(0.8%)	1	(4.2%)	5	(6.6%)	2	(1.7%)	1	(1.7%)	10		(2.5%)	
Chronic obstructive bronchitis, emphysema	0	(0.0%)	0	(0.0%)	3	(3.9%)	7	(5.9%)	2	(3.4%)	12		(3.0%)	
Heart failure	2	(1.7%)	1	(4.2%)	1	(1.3%)	2	(1.7%)	4	(6.9%)	10		(2.5%)	
Ischaemic heart disease	2	(1.7%)	10	(41.7%)	8	(10.5%)	4	(3.4%)	1	(1.7%)	25		(6.3%)	
Neurological disease	0	(0.0%)	0	(0.0%)	0	(0.0%)	3	(2.5%)	3	(5.2%)	6		(1.5%)	
Stroke or transient ischemic attack	6	(5.0%)	2	(8.3%)	4	(5.3%)	1	(0.8%)	3	(5.2%)	16		(4.0%)	
Diabetes type I or II	1	(0.8%)	4	(16.7%)	73	(96.1%)	6	(5.0%)	5	(8.6%)	89		(22.4%)	
Oeso-gastroduodenal pathology	0	(0.0%)	3	(12.5%)	4	(5.3%)	5	(4.2%)	8	(13.8%)	20		(5.0%)	
Obesity	0	(0.0%)	2	(8.3%)	30	(39.5%)	2	(1.7%)	8	(13.8%)	42		(10.6%)	
Depression	2	(1.7%)	2	(8.3%)	8	(10.5%)	13	(10.9%)	8	(13.8%)	33		(8.3%)	
Anxiety or panic attacks	0	(0.0%)	2	(8.3%)	3	(3.9%)	8	(6.7%)	4	(6.9%)	17		(4.3%)	
Decreased visual acuity	1	(0.8%)	0	(0.0%)	4	(5.3%)	6	(5.0%)	6	(10.3%)	17		(4.3%)	
Hearing problems	0	(0.0%)	1	(4.2%)	1	(1.3%)	2	(1.7%)	3	(5.2%)	7		(1.8%)	
HIV	0	(0.0%)	0	(0.0%)	0	(0.0%)	108	(90.8%)	0	(0.0%)	108		(27.2%)	

### Acceptability of the questionnaire

The distribution of responses was homogeneous for items 23 to 30 on health and risky consumption and were heterogeneous for items 1 to 22 on medical adherence. Nearly 90% of respondents stated that they followed medical prescriptions perfectly. Therefore, the response modalities for items 1 to 22 were reduced to two modalities (“very good adherence behaviours” versus the three others “more nuanced behaviours”). The most frequently missing items were those concerning forgetting medication, with 20% for item 11 “*I sometimes forget my medication in the afternoon*” and 16.4% for item 10 “*I sometimes forget my midday medication”*. Missing items and ceiling and floor effects of the items are described in Table 2.

**Table 2:** Distribution of GACID-P items responses

				Modalities							
	Items	Missing		Never		2		3		All the time	
		n	(%)	n	(%)	n	(%)	n	(%)	n	(%)
Q1	I take all part of my prescribed medications	2	(0.5)	1	(0.3)	5	(1.3)	25	(6.3)	364	(92.2)
Q2	I take only part of my prescribed medications	37	(9.3)	299	(83.1)	9	(2.5)	13	(3.6)	39	(10.8)
Q3	I take my medication at the prescribed times	7	(1.8)	8	(2.1)	6	(1.5)	88	(22.6)	288	(73.8)
Q4	I comply with the doses prescribed	6	(1.5)	3	(0.8)	2	(0.5)	13	(3.3)	373	(95.4)
Q5	I comply with my doctor’s prescription for how many times a day to take my medication	7	(1.8)	9	(2.3)	5	(1.3)	14	(3.6)	362	(92.8)
Q6	I sometimes change the dose of my medication	12	(3.0)	344	(89.4)	13	(3.4)	11	(2.9)	17	(4.4)
Q6a	I sometimes take more than prescribed	18	(4.5)	349	(92.1)	11	(2.9)	8	(2.1)	11	(2.9)
Q6b	I sometimes take less than the prescribed dose of medication	19	(4.8)	313	(82.8)	32	(8.5)	24	(6.3)	9	(2.4)
Q7	sometimes forget to take my medication	17	(4.3)	238	(62.6)	79	(20.8)	49	(12.9)	14	(3.7)
Q8	There are some medications that I forget to take more than others	12	(3.0)	305	(79.2)	37	(9.6)	23	(6.0)	20	(5.2)
Q9	I sometimes forget my morning medication	35	(8.8)	308	(85.1)	25	(6.9)	14	(3.9)	15	(4.1)
Q10	I sometimes forget my midday medication	65	(16.4)	289	(87.0)	16	(4.8)	15	(4.5)	12	(3.6)
Q11	I sometimes forget my medication in the afternoon	81	(20.4)	289	(91.5)	7	(2.2)	10	(3.2)	10	(3.2)
Q12	I sometimes forget my evening medication	33	(8.3)	282	(77.5)	44	(12.1)	24	(6.6)	14	(3.8)
Q13	I sometimes forget my medication over the week-end	41	(10.3)	300	(84.3)	30	(8.4)	15	(4.2)	11	(3.1)
Q14	I sometimes forget my medication while on vacation	40	(10.1)	290	(81.2)	41	(11.5)	14	(3.9)	12	(3.4)
Q15	I have already voluntarily stopped taking my medication without medical advice	9	(2.3)	329	(84.8)	32	(8.2)	17	(4.4)	10	(2.6)
Q16	On my own initiative, I have already tried to modify my treatment	11	(2.8)	364	(94.3)	5	(1.3)	4	(1.0)	13	(3.4)
Q17	I take my medication for the duration prescribed by my doctor	3	(0.8)	30	(7.6)	10	(2.5)	18	(4.6)	336	(85.3)
Q18	I take my medication according to the instructions	6	(1.5)	5	(1.3)	5	(1.3)	29	(7.4)	352	(90.0)
Q19	I go for the tests prescribed by my doctor (blood, urine tests etc.)	4	(1.0)	1	(0.3)	2	(0.5)	7	(1.8)	383	(97.5)
Q20	I go for the x-ray examinations prescribed by my doctor	6	(1.5)	6	(1.5)	3	(0.8)	8	(2.0)	374	(95.7)
Q21	I attend appointments with my generalist doctor	9	(2.3)	11	(2.8)	2	(0.5)	10	(2.6)	365	(94.1)
Q22	I attend appointments with my specialist doctor	11	(2.8)	2	(0.5)	1	(0.3)	9	(2.3)	374	(96.9)
Q23	I have regular physical activity, suited to my state of health	13	(3.3)	48	(12.5)	82	(21.4)	108	(28.1)	146	(38.0)
Q24	I have a healthy, balanced diet	16	(4.0)	7	(1.8)	45	(11.8)	162	(42.5)	167	(43.8)
Q25	I limit the amount of fat I take	13	(3.3)	32	(8.3)	62	(16.1)	158	(41.1)	132	(34.4)
Q26	I limit the amount of sugar I take	13	(3.3)	37	(9.6)	69	(18.0)	136	(35.4)	142	(37.0)
Q27	I limit the amount of salt I take	10	(2.5)	35	(9.0)	75	(19.4)	129	(33.3)	148	(38.2)
Q28	I limit the amount of alcohol I drink	20	(5.0)	21	(5.6)	37	(9.8)	101	(26.8)	218	(57.8)
Q29	I am smoking less	34	(8.6)	38	(10.5)	36	(9.9)	28	(7.7)	261	(71.9)
Q30	I allow myself sufficient resting periods (working)	10	(2.5)	15	(3.9)	58	(15.0)	113	(29.2)	201	(51.9)

### Factorial structure

PCA with a Promax oblique rotation performed on polytomic items revealed two groups: items 25 to 29 under the dimension “**Limitation of risk-related consumer habits**” and items 23, 24 and 30 under the dimension “**Healthy lifestyle**”.

The MCA of re-coded dichotomous items 1 to 22 identified a first factor, **“Forgetting to take medication**” (items 6b and 8 to 14). The three other factors were not clearly distinguishable from each other, as many items loaded on several of them. Therefore, we decided to group them into a single dimension **“Intention to comply with treatment**” (items 1 to 6b and 16 to 22). Items 7 and 15 loaded very little on the factors. In addition, the generic nature of item 7 did not add much to the measure, given the number of other items in the same field. And for item 15, the meaning of “voluntarily” could be ambiguous. They were therefore removed from the questionnaire. MCA results are in supplementary files (Table 1 S).

### Results of the IRT analysis

The IRT analysis involved the four dimensions defined above.

“Limitation of risk-related consumer habits” dimension (5 items).

**Fig. 2:**
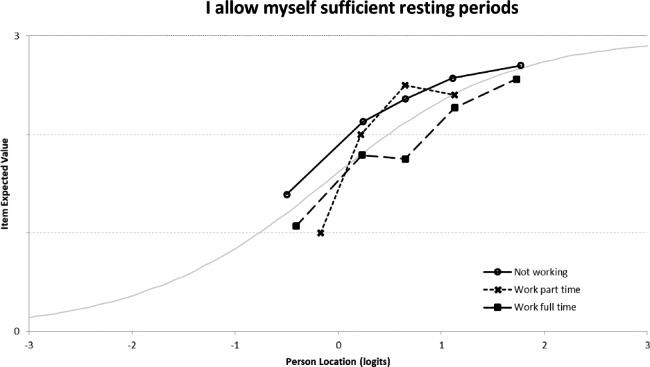
Differential item functioning graph of the smoking status for item “I am smoking less”

The PSI was reasonable (PSI = 0.65). The results did not show any misfit of items or individuals. The threshold map showed an inversion of the thresholds for item 29 (*I am smoking less)* that was not perceived in the same way by smokers and non-smokers (or former smokers) with regard to the latent trait (presence of DIF, p < 0.0001) (Fig. 2). An optimized version of this dimension was defined by creating a conditioned QF29 item completed only by smoking individuals, which left the PSI unchanged (PSI = 0.66).

“Healthy lifestyle” dimension (3 items).

**Fig. 3:**
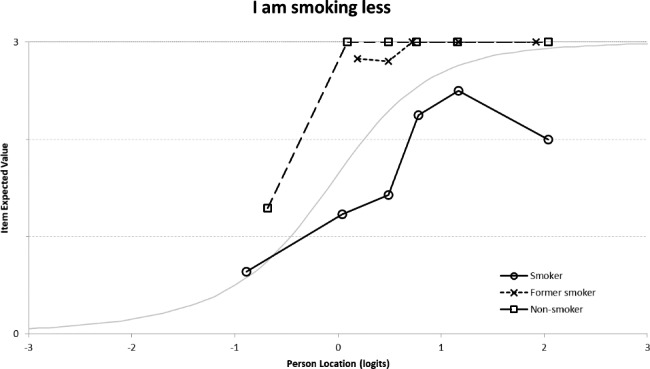
Differential item functioning graph of the professional status for item “I allow myself sufficient resting periods”

The PSI was very low (PSI = 0.30). The results did not show any misfit of items or individuals. The threshold map showed ordered thresholds for the three items. A uniform DIF was found on item 30 (*I allow myself sufficient resting periods)* (p = 0.001) depending on the presence or absence of a professional activity (Fig. 3). This item was split into two groups: those who work and those who do not. The threshold map revealed that at the same level of the latent trait “**Healthy lifestyle**”, a person who worked tended to give less time for sufficient rest.

“Forgetting to take medication” dimension (8 items).

PSI was very low (PSI = 0.20), which can be explained by a discrepancy in the latent trait between those who completed the questionnaire and the difficulty of the items themselves (mean − 0.684 [SD 0.869]). Two items (items 6b “*I sometimes take less than the prescribed dose of medication*” and 8 “*There are some medications that I forget to take more than other*s”) were poorly adjusted with an overfit (i.e., were too discriminating and had redundancies). The threshold map showed dichotomous prescribed thresholds. Items 10 “*I sometimes forget my midday medication*” and 11 *“Sometimes I forget my medication in the afternoon*” also showed a high correlation of their residuals (r = 0.38). We decided to delete item 11, which presented a higher number of missing data and also a higher location (Location = 1.18) with a strong floor effect (91.5%) because medication is rarely taken in the afternoon. However, despite the optimization of this dimension to seven items, the PSI decreased to 0.11.

“Intention to comply with treatment” dimension (14 items).

The PSI of -0.10 indicated that the items were not sufficiently “difficult” and therefore could not discriminate between the most and least observant patients, knowing that these patients with chronic diseases are themselves more apt to have good adherence. Moreover, 82% of participants answered “All the time” to almost all the questions answered.

This poor PSI led to poor adjustments of items and individuals, such as for three items (items 1 “*I take all part of my prescribed medications*”, 4 *“I comply with the doses prescribed*” and 6 *“I sometimes change the dose of my medication*”) that presented an underfit. After examining the content, we decided to delete item 1, which presented an underfit with a residual fit of -2.4 (p = 0.009), explained mainly by strong local dependency with item 2 *“I take only part of my prescribed medications”* (r = 0.29). The same reasoning occurred for item 6, which presented poor fit and strong local dependency with items 6a “*I sometimes take more than prescribed*” (r = 0.26) and 16 “*On my own initiative, I have already tried to modify my treatment*”. We decided to keep item 6 and delete items 6a and 16. Similarly, we deleted item 18 *“I take my medication according to the instructions*”, which was considered too similar to item 3 *“I take my medication at the prescribed times*”. Therefore, the dimension was reduced by four items (deletion of items 1, 6a, 16, 18).

Table 3 shows the results of the IRT analyses for all dimensions optimized by the results of the statistical analysis and recommendations of the scientific committee.

**Table 3:** Summary of the results of the item and scale modalities analysis (Rasch and Partial Credit Model analyses) of the French Generic Adherence for Chronic Diseases Profile (GACID-P) optimized dimensions

Items per dimension		Location	SE	Fit Residual	P value
**Limitation of risk-related consumer habits** (PSI = 0.66) (PSI = 0,66)					
Q25	I limit the amount of fat I take	0.03	0.09	-1.10	0.17
Q26	I limit the amount of sugar I take	0.08	0.08	-0.96	0.11
Q27	I limit the amount of salt I take	-0.03	0.08	-1.55	0.01
Q28	I limit the amount of alcohol I drink	-0.54	0.08	2.72	< 0.001
QF29	I am smoking less *(for smokers)*	0.46	0.21	0.95	0.92
**Healthy lifestyle** (PSI = 0.29)					
Q23	I have regular physical activity, suited to my state of health	0.53	0.07	-0.58	0.05
Q24	I have a healthy, balanced diet	-0.38	0.09	0.85	0.50
Q30T	I allow myself sufficient resting periods (working)	0.16	0.13	0.45	0.78
Q30NT	I allow myself sufficient resting periods (not working)	-0.31	0.09	1.28	0.38
**Forgetting to take medication** (PSI = 0.11)					
Q6b	I sometimes take less than the prescribed dose of medication	0.04	0.19	2.30	0.25
Q8	There are some medications that I forget to take more than others	-0.25	0.18	2.29	0.79
Q9	I sometimes forget my morning medication	0.24	0.21	0.31	0.45
Q10	I sometimes forget my midday medication	0.42	0.22	0.40	0.50
Q12	I sometimes forget my evening medication	-0.43	0.19	1.37	0.22
Q13	I sometimes forget my medication over the week-end	0.23	0.20	-1.87	0.002
Q14	I sometimes forget my medication while on vacation	-0.26	0.20	0.04	0.35
**Intention to comply with treatment** (PSI=-0.38)					
Q2	I take only part of my prescribed medications	1.09	0.17	1.43	0.68
Q3	I take my medication at the prescribed times	1.76	0.15	2.91	0.63
Q4	I comply with the doses prescribed	-0.71	0.25	-2.21	0.01
Q5	I comply with my doctor’s prescription for how many times a day to take my medication and how many days to take my medication	-0.09	0.21	-1.56	0.06
Q6	I sometimes change the dose of my medication	0.55	0.18	-1.15	0.04
Q17	I take my medication for the duration prescribed by my doctor	0.90	0.16	-0.33	0.41
Q19	I go for the tests prescribed by my doctor (blood, urine tests etc.)	-1.50	0.34	-0.75	0.33
Q20	I go for the x-ray examinations prescribed by my doctor	-0.72	0.25	-0.83	0.43
Q21	I attend appointments with my generalist doctor	-0.14	0.21	-0.67	0.16
Q22	I attend appointments with my specialist doctor	-1.15	0.30	-1.87	0.14

PSI: person separation index ; SE : Standard Error

### Results of classical test theory analysis

Table 4 shows the results of classical test theory calculated for the four dimensions. Cronbach’s alpha coefficients were acceptable. ICC coefficients showed average reproducibility at 15 days. Convergence of the GACID-P dimension scores with the MMAS-8 score was moderate. The lower Spearman correlation coefficients for **“Forgetting to take medication”** (0.62) and **“Intention to comply with treatment”** (0.41) optimized dimensions are explained by the latter dimension consisting of items not related to medication adherence (performing tests; attending doctor’s appointments), whereas the former consists exclusively of items asking about forgetting to take medication.

**Table 4:** Summary of the results of the validity analyses (according to classical theory of GACID-P optimized dimensions)

Dimension	Cronbach’s alpha	N	ICC (95% CI)	
Limitation of risk-related consumer habits	0.82	308	0.55 (0.47–0.62)	
Healthy lifestyle	0.62	311	0.66 (0.59–0.72)	
Forgetting to take medication	0.85	312	0.64 (0.57–0.71)	
Intention to comply with treatment	0.65	314	0.49 (0.40–0.57)	

*Reproducibility was assessed between day 0 and day 15 by the intraclass correlation coefficient (ICC) derived from a mixed ANOVA model, CI: confidence interval*

### Scoring

The statistical analyses showed DIF for one item in the dimensions “**Limitation of risk-related consumer habit**s” by smoking status and “**Healthy lifestyle**” by professional status. Therefore, the score for these 2 dimensions cannot be calculated in the same way according to the category of these variables. A weighted score for the dimension “**Limitation of risk-related consumer habit**s” was calculated for each smoking status category (i.e. smoker vs. non-smoker) and similarly for the dimension “**Healthy lifestyle**” according to professional status (i.e. the worker vs. non-worker)Therefore, a table of correspondence between the sum of the items completed and a weighted score, representing the position on latent trait, was recommended for these two dimensions. For the two other dimensions, the use of the crude score, (i.e.the mean score of the completed items multiplied by the total number of items in the dimension) was recommended, having first reduced the four modalities of response to the items to two modalities (“very good adherence behaviours” versus the three others, “more nuanced behaviours”). To facilitate their interpretation, these weighted and crude scores were then linearized from 0 (poorer adherence) to 10 (better adherence), except for the “**Forgetting to take medication**”, with reversed interpretation.

We recommend that if more than one item is missing, the score for the dimension cannot be calculated.

Tables 2S to 5S in supplementary filecorresponds to the manual of scores calculated for the four dimensions.

## Discussion

This work describes the development of a questionnaire from its preliminary phases of item development, then reduction and optimization to obtain a questionnaire of 25 items in four dimensions adapted to measuring the generic phenomenon of adherence in chronic diseases. The exploratory analysis revealed four dimensions of adherence, beyond the three initial dimensions proposed during the development phase. The final version of the French GACID-P scale consists of 25 items with four response modalities, including one item conditioned on tobacco use. One score per dimension is calculated to obtain four adherence profile scores that can be easily integrated into the patient care pathway. This score corresponds to the sum of the items for the dimensions “**Forgetting to take medication**” and “**Intention to comply with treatment**” and to a weighted score according to the IRT analysis for the dimensions “**Limitation of risk-related consumer habits**” and “**Healthy lifestyle”.**

Two dimensions with polytomous items (“**Limitation of risk-related consumer habits**” and “**Healthy lifestyle**”) cover the field of adherence to a healthy lifestyle and diet, without showing any maladjustment of items or person in IRT analysis. Nevertheless, we decided that item 29 should be completed only by smokers and to consider the professional status (working vs. not working) when analysing the results of item 30. Thus, for these two dimensions, the use of weighted scores from the IRT analysis is recommended.

The two other dimensions with dichotomous items (“**Forgetting to take medication**” and “**Intention to comply with treatment**”) cover medication and/or medical adherence. For the “**Forgetting to take medication**” dimension, we observed a discrepancy on the latent trait between the participants’ ability to complete the items and the difficulty of the items themselves. In fact, when dealing with chronically ill people, the people responding to the questionnaire are probably used to taking their medication and therefore forgetting is less common in our sample than it would be in a more general population.

The WHO considers that improving adherence to chronic treatment would have greater impact on human health than the development of new medical therapies [31]. In addition to the impact on the daily life of patients, the consequences are heavy in financial terms, and non-adherence also complicates the relationship with the doctor and can lead to a poor evaluation of the patient’s state of health. Therefore, having a tool that allows for obtaining four different adherence profiles is an asset for research. Indeed, some researchers will be interested in this or that dimension more particularly, depending on their research question. The scale will also be of interest for the clinic, its objective being to improve the health status of individuals and populations and to encourage appropriate behaviour. In addition, we chose to tolerate only one missing item for calculating weighted scores. This decision will require investigators to be more rigorous or to ease the completion of the questionnaire by its presentation on apps in order to avoid missing items as much as possible. Another strength of this study, in addition to the originality of the questionnaire developed and validated, is the nature of the sample with several chronic diseases and the results not showing any difference in the understanding of the items whatever the condition.

Our study has some limitations. For logistical reasons, the questionnaires were completed by patients just before the consultation, then the doctor checked whether the questionnaire was complete during the consultation. One may wonder about the existence of a possible measurement bias due to a “white-coat” effect. Patients may have guessed that their doctor was going to check the questionnaire and therefore more concerned about completing it “correctly” so as not to disappoint their doctor and perhaps could not freely write that they did not comply with the doctor’s prescriptions all the time. Indeed, more generally, patients tend to respond to what their doctors want to hear and therefore overestimate their adherence [32]. Further studies may be needed to make the administration procedure more anonymous (e.g., using a closed, anonymous box to collect the questionnaire outside the physician’s office). Concerning the target population, it is a chronically ill population, which implies long-term treatment and therefore a certain habit of being more observant [33]. The particularity of this population could also explain the results of the dimensions that cover medication adherence. These reasons may explain that our data showed a shift between person abilities and items difficulties which may explain discrepancies in reliability indicators results (PSI and Cronbach’s alpha). Indeed, according to Anselmi et al.: “Classical and modern measures are expected to be substantially the same when the score distribution is symmetric, whereas they are expected to differ more and more with the increasing of the skewness of the score distribution.” [34]. It will be relevant to retest this questionnaire within a general population whose difficulty of response to items would be different from our sample. Another methodological limitation is that this time, the factor analysis was partly dictated by the modalities of response and not the dimensions and therefore the latent trait. Nonetheless, a match between the initial dimensioning and response modalities was respected.

In conclusion, the GACID-P is a French generic scale developed to measure adherence in several disease areas and validated in cardiology, rheumatology, diabetes, cancer and infectiology. Its validity was documented by a theoretical approach and content analysis as well as careful structure validity and scale calibration. It is now available for research targeting adherence in a broad perspective.

## Electronic supplementary material

Below is the link to the electronic supplementary material.

Supplementary Material 1

Table 1S: Results of multiple correspondence analysis of items 1 to 22. Table 2S: Manual for calculating the scores of the dimensions “Intention to comply with treatment” and “Forgetting to take medication. Table 3S: Manual for calculating the scores of “Healthy lifestyle” and “Limitation of consumption at risk” dimensions. Table 4S: Manual for calculating the scores of “Limitation of consumption at risk” dimension if one item is missing. Table 5S: Manual for calculating the scores of “Healthy lifestyle” dimension if one item is missing.
