# Supplementary material for: Validation and optimization of the French Generic Adherence for Chronic Diseases Profile (GACID-P) using classical test and item response theory
Source: Health Qual Life Outcomes. 2023 May 24;21:49. doi: 10.1186/s12955-023-02130-0 (PMC10210406; doi:10.1186/s12955-023-02130-0)
Supplement: Supplementary file 1 — Supplementary Material 1 [file 12955_2023_2130_MOESM1_ESM.docx]

**Questionnaire GACID - P**

**Generic adherence for chronic diseases profile**

**Please read the following instructions carefully:**

- Check the box that best describes your situation between "**never**" and "**always**",

according to the proposed sentences

- There is no right or wrong answer
- For each sentence, circle one box

**Example:**

| **Never Always** | | | | | |
| --- | --- | --- | --- | --- | --- |
| **Q01** | I limit the amount of fat I take | 1 | 2 | ③ | 4 |

**Filling date: |__|__| |__|__| |__|__|__|__|**

**THIS QUESTIONNAIRE HAS 2 PAGES (WITH THIS ONE).**

| **Limitation of risk-related consumer habits Never Always** | | | | | |
| --- | --- | --- | --- | --- | --- |
| **Q01** | I limit the amount of fat I take | 1 | 2 | 3 | 4 |
| **Q02** | I limit the amount of sugar I take | 1 | 2 | 3 | 4 |
| **Q03** | I limit the amount of salt I take | 1 | 2 | 3 | 4 |
| **Q04** | I limit the amount of alcohol I drink | 1 | 2 | 3 | 4 |
|  | Are you a current smoker? No   *Go to question Q06*  Yes  *Answer to question Q05* | | | | |
| **Q05** | I am smoking less | 1 | 2 | 3 | 4 |
| **Healthy lifestyle Never Always** | | | | | |
| **Q06** | I have regular physical activity, suited to my state of health | 1 | 2 | 3 | 4 |
| **Q07** | I have a healthy, balanced diet | 1 | 2 | 3 | 4 |
|  | I have a full-time job  I have a part-time job  I am unemployed (retired, other) | | | | |
| **Q08** | I allow myself sufficient resting periods | 1 | 2 | 3 | 4 |
| **Forgetting to take medication Never Always** | | | | | |
| **Q09** | I sometimes take less than the prescribed dose of medication | 1 | 2 | 3 | 4 |
| **Q10** | There are some medications that I forget to take more than others | 1 | 2 | 3 | 4 |
| **Q11** | I sometimes forget my morning medication | 1 | 2 | 3 | 4 |
| **Q12** | I sometimes forget my lunchtime medication | 1 | 2 | 3 | 4 |
| **Q13** | I sometimes forget my evening medication | 1 | 2 | 3 | 4 |
| **Q14** | I sometimes forget my medication over the weekend | 1 | 2 | 3 | 4 |
| **Q15** | I sometimes forget my medication while on vacation | 1 | 2 | 3 | 4 |
| **Intention to comply with treatment medication Never Always** | | | | | |
| **Q16** | I take only part of my prescribed medications | 1 | 2 | 3 | 4 |
| **Q17** | I take my medication at the prescribed times | 1 | 2 | 3 | 4 |
| **Q18** | I comply with the doses prescribed | 1 | 2 | 3 | 4 |
| **Q19** | I comply with my doctor's prescription for how many times a day to take my medication | 1 | 2 | 3 | 4 |
| **Q20** | I sometimes change the dose of my medication | 1 | 2 | 3 | 4 |
| **Q21** | I take my medication for the duration prescribed by my doctor | 1 | 2 | 3 | 4 |
| **Q22** | I go for the tests prescribed by my doctor (blood, urine tests, etc) | 1 | 2 | 3 | 4 |
| **Q23** | I go for the x-ray examinations prescribed by my doctor | 1 | 2 | 3 | 4 |
| **Q24** | I attend appointments with my generalist doctor | 1 | 2 | 3 | 4 |
| **Q25** | I attend appointments with my specialist doctor | 1 | 2 | 3 | 4 |
