# Supplementary material for: Validation and optimization of the French Generic Adherence for Chronic Diseases Profile (GACID-P) using classical test and item response theory
Source: Health Qual Life Outcomes. 2023 May 24;21:49. doi: 10.1186/s12955-023-02130-0 (PMC10210406; doi:10.1186/s12955-023-02130-0)
Supplement: Supplementary file 2 — Table 1S: Results of multiple correspondence analysis of items 1 to 22. Table 2S: Manual for calculating the scores of the dimensions “Intention to comply with treatment” and “Forgetting to take medication. Table 3S: Manual for calculating the scores of “Healthy lifestyle” and “Limitation of consumption at risk” dimensions. Table 4S: Manual for calculating the scores of “Limitation of consumption at risk” dimension if one item is missing. Table 5S: Manual for calculating the scores of “Healthy lifestyle” dimension if one item is missing. [file 12955_2023_2130_MOESM2_ESM.docx]

**Journal : Health and Quality of Life Outcomes**

**Title : Validation and optimization of the French Generic Adherence for Chronic Diseases Profile (GACID-P) using classical test and item response theory**

**C. Rotonda ^1,2^, F. Guillemin ^3,4^, T Conroy ^3,5^, C. Alleyrat ^4^, B. Lefevre ^3,6^, M. Soudant ^4^, C. Tarquinio ^1,2^**

^1^ Université de Lorraine, Centre Pierre Janet, F-57000 Metz, France

^2^ Université de Lorraine, APEMAC, F-57000 Metz, France

^3^ Université de Lorraine, APEMAC, F-54000, Nancy, France

^4^ CHRU Nancy, Inserm, Université de Lorraine, CIC, Epidemiologie Clinique, F-54000, Nancy, France;

^5^ Institut de Cancérologie de Lorraine, Department of Medical Oncology, F-54519 Vandœuvre-lès-Nancy, France

^6^ CHRU-Nancy, Université de Lorraine, Service des Maladies Infectieuses et Tropicales, F-54000 Nancy, France

Corresponding author:

Christine Rotonda, PhD

EA4360 APEMAC, Université de Lorraine

lle du Saulcy

57 010 Metz

[christine.rotonda@univ-lorraine.fr](mailto:christine.rotonda@univ-lorraine.fr)

Phone: +33 3 72 74 82 26

<https://orcid.org/0000-0002-8854-0591>

Table 1S: Results of multiple correspondence analysis of items 1 to 22

|  |  | Factor 1 | Factor 2 | | |
| --- | --- | --- | --- | --- | --- |
|  |  |  |  |  |  |
|  |  | **1** | **2** | **3** | **4** |
| Q1 | I take all part of my prescribed medications | 0.037 | 0.038 | 0.074 | 0.092 |
| Q2 | I take only part of my prescribed medications | 0.023 | 0.006 | 0.039 | 0.089 |
| Q3 | I take my medication at the prescribed times | 0.031 | 0.007 | 0.030 | 0.077 |
| Q4 | I comply with the doses prescribed | 0.025 | 0.118 | <0.001 | <0.001 |
| Q5 | I comply with my doctor's prescription for how many times a day to take my medication | 0.025 | 0.094 | 0.048 | 0.004 |
| Q6 | I sometimes change the dose of my medication | 0.024 | 0.014 | 0.160 | 0.007 |
| Q6a | I sometimes take more than prescribed | 0.036 | <0.001 | 0.193 | 0.001 |
| Q6b | I sometimes take less than the prescribed dose of medication | 0.059 | 0.011 | <0.001 | 0.034 |
| Q7 | I sometimes forget to take my medication | 0.046 | 0.000 | 0.015 | 0.045 |
| Q8 | There are some medications that I forget to take more than others | 0.061 | 0.002 | 0.009 | 0.003 |
| Q9 | I sometimes forget my morning medication | 0.047 | 0.053 | 0.005 | 0.002 |
| Q10 | I sometimes forget my midday medication | 0.065 | 0.066 | 0.002 | 0.015 |
| Q11 | I sometimes forget my medication in the afternoon | 0.069 | 0.081 | 0.004 | 0.009 |
| Q12 | I sometimes forget my evening medication | 0.067 | <0.001 | 0.004 | 0.035 |
| Q13 | I sometimes forget my medication over the week-end | 0.072 | 0.044 | <0.001 | 0.017 |
| Q14 | I sometimes forget my medication while on vacation | 0.065 | 0.016 | 0.011 | 0.018 |
| Q15 | I have already voluntarily stopped taking my medication without medical advice | 0.041 | 0.000 | 0.000 | 0.024 |
| Q16 | On my own initiative, I have already tried to modify my treatment | 0.019 | 0.029 | 0.119 | 0.020 |
| Q17 | I take my medication for the duration prescribed by my doctor | 0.015 | 0.042 | 0.016 | 0.073 |
| Q18 | I take my medication according to the instructions | 0.018 | 0.039 | 0.004 | 0.126 |
| Q19 | I go for the tests prescribed by my doctor (blood, urine tests etc) | 0.013 | 0.037 | 0.056 | 0.051 |
| Q20 | I go for the x-ray examinations prescribed by my doctor | 0.003 | 0.067 | 0.030 | 0.105 |
| Q21 | I attend appointments with my generalist doctor | <0.001 | 0.088 | 0.067 | 0.027 |
| Q22 | I attend appointments with my specialist doctor | 0.007 | 0.073 | 0.033 | 0.005 |

Table 2S: Manual for calculating the scores of the dimensions “Intention to comply with treatment” and “Forgetting to take medication”

| **Items per dimension** | **Modalities** | **Initial coding** | **Re-coding** | **C_WL_** | **C_BL_** | **N_WL_** | **N_BL_** |
| --- | --- | --- | --- | --- | --- | --- | --- |
| **Intention to comply with treatment** |  |  |  |  |  |  |  |
| Q2 – I take only part of my prescribed medications | Never | 1 | 1 | 0 | 10 | 0 | 10 |
|  | 2 | 2 | 0 |  |  |  |  |
|  | 3 | 3 | 0 |  |  |  |  |
|  | All the time | 4 | 0 |  |  |  |  |
| Q3 – I take my medication at the prescribed times | Never | 1 | 0 |  |  |  |  |
|  | 2 | 2 | 0 |  |  |  |  |
|  | 3 | 3 | 0 |  |  |  |  |
|  | All the time | 4 | 1 |  |  |  |  |
| Q4 – I comply with the doses prescribed | Never | 1 | 0 |  |  |  |  |
|  | 2 | 2 | 0 |  |  |  |  |
|  | 3 | 3 | 0 |  |  |  |  |
|  | All the time | 4 | 1 |  |  |  |  |
| Q5 – I comply with my doctor's prescription for how many times a day and how many days to take my medication | Never | 1 | 0 |  |  |  |  |
|  | 2 | 2 | 0 |  |  |  |  |
|  | 3 | 3 | 0 |  |  |  |  |
|  | All the time | 4 | 1 |  |  |  |  |
| Q6 – I sometimes change the dose of my medication | Never | 1 | 1 |  |  |  |  |
|  | 2 | 2 | 0 |  |  |  |  |
|  | 3 | 3 | 0 |  |  |  |  |
|  | All the time | 4 | 0 |  |  |  |  |
| Q17 – I take my medication for the duration prescribed by my doctor | Never | 1 | 0 |  |  |  |  |
|  | 2 | 2 | 0 |  |  |  |  |
|  | 3 | 3 | 0 |  |  |  |  |
|  | All the time | 4 | 1 |  |  |  |  |
| Q19 – I go for the tests prescribed by my doctor (blood, urine tests etc) | Never | 1 | 0 |  |  |  |  |
|  | 2 | 2 | 0 |  |  |  |  |
|  | 3 | 3 | 0 |  |  |  |  |
|  | All the time | 4 | 1 |  |  |  |  |
| Q20 – I go for the x-ray examinations prescribed by my doctor | Never | 1 | 0 |  |  |  |  |
|  | 2 | 2 | 0 |  |  |  |  |
|  | 3 | 3 | 0 |  |  |  |  |
|  | All the time | 4 | 1 |  |  |  |  |
| Q21 – I attend appointments with my general practitioner | Never | 1 | 0 |  |  |  |  |
|  | 2 | 2 | 0 |  |  |  |  |
|  | 3 | 3 | 0 |  |  |  |  |
|  | All the time | 4 | 1 |  |  |  |  |
| Q22 – I attend appointments with my specialist doctor | Never | 1 | 0 |  |  |  |  |
|  | 2 | 2 | 0 |  |  |  |  |
|  | 3 | 3 | 0 |  |  |  |  |
|  | All the time | 4 | 1 |  |  |  |  |
| **Forgetting to take medication** |  |  |  |  |  |  |  |
| Q6b – I sometimes take less than the prescribed dose of medication | Never | 1 | 1 | 0 | 7 | 10 | 0 |
|  | 2 | 2 | 0 |  |  |  |  |
|  | 3 | 3 | 0 |  |  |  |  |
|  | All the time | 4 | 0 |  |  |  |  |
| Q8 – There are some medications that I forget to take more than others | Never | 1 | 1 |  |  |  |  |
|  | 2 | 2 | 0 |  |  |  |  |
|  | 3 | 3 | 0 |  |  |  |  |
|  | All the time | 4 | 0 |  |  |  |  |
| Q9 – I sometimes forget my morning medication | Never | 1 | 1 |  |  |  |  |
|  | 2 | 2 | 0 |  |  |  |  |
|  | 3 | 3 | 0 |  |  |  |  |
|  | All the time | 4 | 0 |  |  |  |  |
| Q10 – I sometimes forget my midday medication | Never | 1 | 1 |  |  |  |  |
|  | 2 | 2 | 0 |  |  |  |  |
|  | 3 | 3 | 0 |  |  |  |  |
|  | All the time | 4 | 0 |  |  |  |  |
| Q12 – I sometimes forget my evening medication | Never | 1 | 1 |  |  |  |  |
|  | 2 | 2 | 0 |  |  |  |  |
|  | 3 | 3 | 0 |  |  |  |  |
|  | All the time | 4 | 0 |  |  |  |  |
| Q13 – I sometimes forget my medication over the week-end | Never | 1 | 1 |  |  |  |  |
|  | 2 | 2 | 0 |  |  |  |  |
|  | 3 | 3 | 0 |  |  |  |  |
|  | All the time | 4 | 0 |  |  |  |  |
| Q14 – I sometimes forget my medication when on holiday | Never | 1 | 1 |  |  |  |  |
|  | 2 | 2 | 0 |  |  |  |  |
|  | 3 | 3 | 0 |  |  |  |  |
|  | All the time | 4 | 0 |  |  |  |  |

Calculation of crude score (CS) taking into account missing data: CS = mean of non-missing items*total number of items. Warning: Crude score cannot be calculated if more than one item is missing.

Calculation of normalized score (NS) from 0 to 100 : NS = {[(CS-C_WL_) /(C_BL_-C_WL_)]*(N_BL_-N_WL_)}+N_WL_

C_WL_ et C_BL_ means adherence worst limit and adherence best limit, respectively, of crude score (CS).

N_WL_ et N_BL_ means adherence worst limit and adherence best limit, respectively, of normalized score (NS).

Table 3S: Manual for calculating the scores of “Healthy lifestyle” and “Limitation of consumption at risk” dimensions.

| **Items** | **Modalities** | **Initial Coding** | **Re-coding** | **CS** | **WS if not working** | **CS** | **WS if working** |
| --- | --- | --- | --- | --- | --- | --- | --- |
| **Healthy lifestyle** | | | | | | | |
| Q23 - I have regular physical activity, suited to my state of health | Never | 1 | 0 |  |  | | |
|  | 2 | 2 | 1 | 0 | 0.2 | 0 | 0.0 |
|  | 3 | 3 | 2 | 1 | 1.7 | 1 | 1.6 |
|  | All the time | 4 | 3 | 2 | 2.8 | 2 | 2.9 |
| Q24 - I have a healthy, balanced diet | Never | 1 | 0 | 3 | 3.7 | 3 | 3.9 |
|  | 2 | 2 | 1 | 4 | 4.4 | 4 | 4.8 |
|  | 3 | 3 | 2 | 5 | 5.1 | 5 | 5.6 |
|  | All the time | 4 | 3 | 6 | 5.9 | 6 | 6.4 |
| Q30 - I allow myself sufficient resting periods | Never | 1 | 0 | 7 | 6.8 | 7 | 7.3 |
|  | 2 | 2 | 1 | 8 | 8.0 | 8 | 8.5 |
|  | 3 | 3 | 2 | 9 | 9.6 | 9 | 10.0 |
|  | All the time | 4 | 3 |  |  | | |
| **Items** | **Modalities** | **Initial Coding** | **Re-coding** | **CS** | **WS if non smokers** | **CS** | **WS if smokers** |
| **Limitation of risk-related consumer habits** | | | | | | | |
| Q25 - I limit the amount of fat I take | Never | 1 | 0 |  |  |  |  |
|  | 2 | 2 | 1 | 0 | 0.2 | 0 | 0.0 |
|  | 3 | 3 | 2 | 1 | 1.4 | 1 | 1.2 |
|  | All the time | 4 | 3 | 2 | 2.3 | 2 | 2.0 |
| Q26 - I limit the amount of sugar I take | Never | 1 | 0 | 3 | 2.9 | 3 | 2.6 |
|  | 2 | 2 | 1 | 4 | 3.4 | 4 | 3.1 |
|  | 3 | 3 | 2 | 5 | 3.9 | 5 | 3.6 |
|  | All the time | 4 | 3 | 6 | 4.4 | 6 | 4.0 |
| Q27 - I limit the amount of salt I take l | Never | 1 | 0 | 7 | 4.9 | 7 | 4.4 |
|  | 2 | 2 | 1 | 8 | 5.5 | 8 | 4.8 |
|  | 3 | 3 | 2 | 9 | 6.2 | 9 | 5.3 |
|  | All the time | 4 | 3 | 10 | 7.1 | 10 | 5.8 |
| Q28 - I limit the amount of alcohol I drink | Never | 1 | 0 | 11 | 8.2 | 11 | 6.3 |
|  | 2 | 2 | 1 | 12 | 9.6 | 12 | 6.9 |
|  | 3 | 3 | 2 |  |  | 13 | 7.7 |
|  | All the time | 4 | 3 |  |  | 14 | 8.7 |
| Q29F - I am smoking less *(for smokers)* | Never | 1 | 0 |  |  | 15 | 10.0 |
|  | 2 | 2 | 1 |  |  |  |  |
|  | 3 | 3 | 2 |  |  |  |  |
|  | All the time | 4 | 3 |  |  |  |  |

CS: crude score (from 0 to 9 for “healthy lifestyle” dimension, from 0 to 12 for the “Limitation of consumption at risk” dimension and for non-smokers and from 0 to 15 for the “Limitation of consumption at risk” dimension and for smokers); WS: weighted score. The WS was calculated if all items in the dimension are completed.

Table 4S: Manual for calculating the scores of “Limitation of consumption at risk” dimension if one item is missing.

| **Items** | **Modalities** | **Initial coding** | **Re-coding** | **CS** | **WS if non-smoker** | | | | | | | **CS** | **WS if smoker** | | | | | | | | |
| --- | --- | --- | --- | --- | --- | --- | --- | --- | --- | --- | --- | --- | --- | --- | --- | --- | --- | --- | --- | --- | --- |
|  |  |  |  |  | if missing item is: | | | | | | |  | if missing item is: | | | | | | | | |
|  |  |  |  |  | *Q25* | | *Q26* | | *Q27* | *Q28* | |  | | *Q25* | *Q26* | *Q27* | | *Q28* | | *Q29F* | |
| Q25 - I limit the amount of fat I take | Never | 1 | 0 |  |  |  | |  | | |  |  |  | |  | |  | |  | |  |
|  | 2 | 2 | 1 | 0 | 0.7 | 0.6 | | 0.8 | | | 0.5 | 0 | 0.4 | | 0.3 | | 0.5 | | 0.2 | | 0.2 |
|  | 3 | 3 | 2 | 1 | 1.9 | 1.8 | | 1.9 | | | 1.8 | 1 | 1.6 | | 1.5 | | 1.6 | | 1.5 | | 1.4 |
|  | All the time | 4 | 3 | 2 | 2.8 | 2.7 | | 2.7 | | | 2.8 | 2 | 2.5 | | 2.4 | | 2.4 | | 2.4 | | 2.3 |
| Q26 - I limit the amount of sugar I take | Never | 1 | 0 | 3 | 3.4 | 3.3 | | 3.4 | | | 3.6 | 3 | 3.1 | | 3.0 | | 3.0 | | 3.1 | | 2.9 |
|  | 2 | 2 | 1 | 4 | 4.1 | 3.9 | | 4.0 | | | 4.3 | 4 | 3.6 | | 3.5 | | 3.6 | | 3.7 | | 3.4 |
|  | 3 | 3 | 2 | 5 | 4.7 | 4.6 | | 4.6 | | | 5.1 | 5 | 4.1 | | 4.0 | | 4.0 | | 4.3 | | 3.9 |
|  | All the time | 4 | 3 | 6 | 5.4 | 5.3 | | 5.4 | | | 5.9 | 6 | 4.6 | | 4.5 | | 4.6 | | 4.9 | | 4.4 |
| Q27 - I limit the amount of salt I take | Never | 1 | 0 | 7 | 6.3 | 6.3 | | 6.3 | | | 6.8 | 7 | 5.1 | | 5.1 | | 5.1 | | 5.5 | | 4.9 |
|  | 2 | 2 | 1 | 8 | 7.4 | 7.5 | | 7.6 | | | 8.0 | 8 | 5.7 | | 5.7 | | 5.7 | | 6.1 | | 5.5 |
|  | 3 | 3 | 2 | 9 | 8.7 | 9.0 | | 9.1 | | | 9.4 | 9 | 6.4 | | 6.4 | | 6.4 | | 6.8 | | 6.2 |
|  | All the time | 4 | 3 |  |  |  | |  | | |  | 10 | 7.1 | | 7.2 | | 7.2 | | 7.5 | | 7.1 |
| Q28 - I limit the amount of alcohol I drink | Never | 1 | 0 |  |  |  | |  | | |  | 11 | 8.1 | | 8.3 | | 8.3 | | 8.6 | | 8.2 |
|  | 2 | 2 | 1 |  |  |  | |  | | |  | 12 | 9.5 | | 9.7 | | 9.7 | | 9.9 | | 9.6 |
|  | 3 | 3 | 2 |  |  |  | |  | | |  |  |  | |  | |  | |  | |  |
|  | All the time | 4 | 3 |  |  |  | |  | | |  |  |  | |  | |  | |  | |  |
| Q29F - I am smoking less (for smokers) | Never | 1 | 0 |  |  |  | |  | | |  |  |  | |  | |  | |  | |  |
|  | 2 | 2 | 1 |  |  |  | |  | | |  |  |  | |  | |  | |  | |  |
|  | 3 | 3 | 2 |  |  |  | |  | | |  |  |  | |  | |  | |  | |  |
|  | All the time | 4 | 3 |  |  |  | |  | | |  |  |  | |  | |  | |  | |  |

CS: crude score (from 0 to 9 for non-smoker and with one item missing and from 0 to 12 for smoker and with one item missing); WS: weighted score. The WS was calculated if one item in the dimension is missing.

Table 5S: Manual for calculating the scores of “Healthy lifestyle” dimension if one item is missing.

| **Items** | **Modalities** | **Initial coding** | **Re-coding** | **CS** | **WS if not working** | | | **CS** | **WS if working** | | |
| --- | --- | --- | --- | --- | --- | --- | --- | --- | --- | --- | --- |
|  |  |  |  |  | if missing item is : | | |  | if missing item is : | | |
|  |  |  |  |  | *Q23* | *Q24* | *Q30* |  | *Q23* | *Q24* | *Q30* |
| Q23 - I have regular physical activity, suited to my state of health | Never | 1 | 0 |  |  |  |  |  |  |  |  |
|  | 2 | 2 | 1 | 0 | 0.4 | 1.8 | 0.6 | 0 | 0.2 | 1.6 | 0.6 |
|  | 3 | 3 | 2 | 1 | 1.9 | 3.1 | 2.3 | 1 | 1.8 | 3.3 | 2.3 |
|  | All the time | 4 | 3 | 2 | 3.1 | 4.1 | 3.9 | 2 | 3.3 | 4.6 | 3.9 |
| Q24 - I have a healthy. balanced diet | Never | 1 | 0 | 3 | 4.2 | 5.0 | 5.1 | 3 | 4.7 | 5.6 | 5.1 |
|  | 2 | 2 | 1 | 4 | 5.2 | 5.9 | 6.2 | 4 | 6.1 | 6.5 | 6.2 |
|  | 3 | 3 | 2 | 5 | 6.6 | 7.1 | 7.6 | 5 | 7.6 | 7.7 | 7.6 |
|  | All the time | 4 | 3 | 6 | 8.6 | 8.6 | 9.3 | 6 | 9.3 | 9.3 | 9.3 |
| Q30 - I allow myself sufficient resting periods | Never | 1 | 0 |  |  |  |  |  |  |  |  |
|  | 2 | 2 | 1 |  |  |  |  |  |  |  |  |
|  | 3 | 3 | 2 |  |  |  |  |  |  |  |  |
|  | All the time | 4 | 3 |  |  |  |  |  |  |  |  |

CS: crude score (from 0 to 6 with one item missing); WS: weighted score. The WS was calculated if one item in the dimension is missing for working and for not working
